# Tinker, Tailor, Tumour Suppressor: The Many Functions of PRP4K

**DOI:** 10.3389/fgene.2022.839963

**Published:** 2022-02-24

**Authors:** Elias B. Habib, Sabateeshan Mathavarajah, Graham Dellaire

**Affiliations:** ^1^ Dalhousie University, Department of Pathology, Halifax, NS, Canada; ^2^ Department of Biochemistry and Molecular Biology, Dalhousie University, Halifax, NS, Canada; ^3^ Beatrice Hunter Cancer Research Institute, Halifax, NS, Canada

**Keywords:** pre-mRNA processing factor 4 kinase (PRP4K), pre-mRNA splicing, spindle assembly checkpoint (SAC), yes-associated protein (YAP), epidermal growth factor receptor (EGFR), taxane resistance, anoikis, tumour suppressor

## Abstract

Pre-mRNA processing factor 4 kinase (PRP4K, also known as PRPF4B) is an essential kinase first identified in the fission yeast *Schizosaccharomyces pombe* that is evolutionarily conserved from amoebae to animals. During spliceosomal assembly, PRP4K interacts with and phosphorylates PRPF6 and PRPF31 to facilitate the formation of the spliceosome B complex. However, over the past decade additional evidence has emerged that PRP4K has many diverse cellular roles beyond splicing that contribute to tumour suppression and chemotherapeutic responses in mammals. For example, PRP4K appears to play roles in regulating transcription and the spindle assembly checkpoint (SAC), a key pathway in maintaining chromosomes stability and the response of cancer cells to taxane-based chemotherapy. In addition, PRP4K has been revealed to be a haploinsufficient tumour suppressor that promotes aggressive cancer phenotypes when partially depleted. PRP4K is regulated by both the HER2 and estrogen receptor, and its partial loss increases resistance to the taxanes in multiple malignancies including cervical, breast and ovarian cancer. Moreover, ovarian and triple negative breast cancer patients harboring tumours with low PRP4K expression exhibit worse overall survival. The depletion of PRP4K also enhances both Yap and epidermal growth factor receptor (EGFR) signaling, the latter promoting anoikis resistance in breast and ovarian cancer. Finally, PRP4K is negatively regulated during epithelial-to-mesenchymal transition (EMT), a process that promotes increased cell motility, drug resistance and cancer metastasis. Thus, as we discuss in this review, PRP4K likely plays evolutionarily conserved roles not only in splicing but in a number of cellular pathways that together contribute to tumour suppression.

## Introduction

**FIGURE 1:**
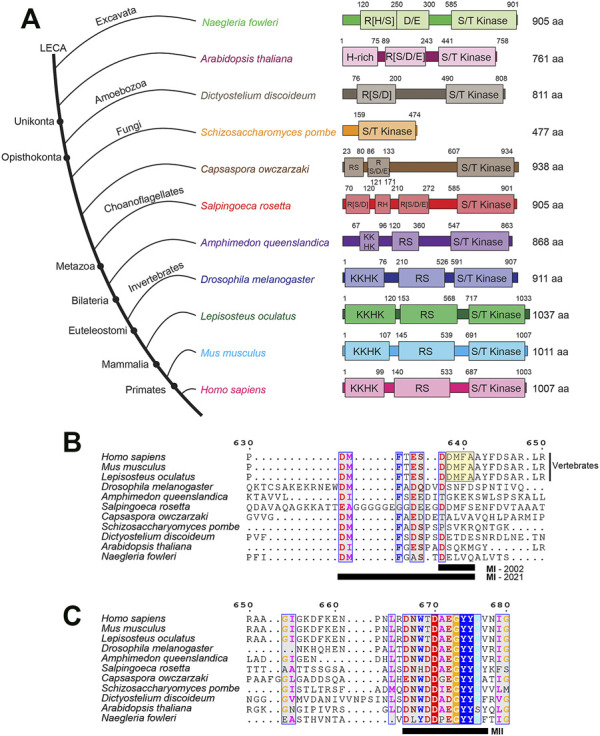
The conserved domain architecture of PRP4K orthologs in representative eukaryotes. **(A)** PRP4K is highly conserved in different eukaryote clades, with orthologs present in representative species of Bikonta (*Naegleria floweri* and *Arabidopsis thaliana*) and Unikonta (*Dictyostelium discoideum*, *Schizosaccharomyces pombe*, *Capsaspora owczarzaki*, *Salpingoeca rosetta*, *Amphimedon queenslandica*, *Drosophila melanogaster*, *Lepisosteus oculatus*, *Mus musculus*, and *Homo sapiens*) (Dellaire et al., 2002). Representative species are shown in the consensus cladogram showing the topology (branch lengths are not intended to describe phylogenetic relationships) (Longhorn et al., 2007; Rodríguez-Ezpeleta et al., 2007; Schierwater et al., 2009; Fritz-Laylin et al., 2010). Domains were predicted using InterPro, with sequence positions presented above the domain (Blum et al., 2021). The kinase domain (shown as S/T kinase, Serine/Threonine kinase) was annotated via InterPro. Whereas, the disordered regions (shown as H-rich, Histidine-rich; KKHK, lysine-histidine repeats; RS, arginine-serine repeats; R [S/D/E], arginine repeats adjacent to serine, aspartate, and glutamate; R [D], arginine-aspartate repeats) were annotated via MobiDB Lite (Necci et al., 2017). Protein sizes for each ortholog are listed in terms of amino acid length to the right of the domain architectures. MUSCLE alignments were performed on PRP4K ortholog sequences (excluding *Arabidopsis thaliana*) to describe the **(B)** MI and **(C)** MII motifs (residues are underlined, black bar) first described by Dellaire et al., 2002. The alignments were visualized using ESPript 3.0 (Robert & Gouet, 2014) and coloured residues indicate different functional groups that are conserved. Dashes indicate gaps within the sequence alignment between orthologs. Positions for the alignment correspond to the human PRP4K protein sequence. Note, that the original MI motif (yellow boxed sequence DDMFA, MI-2002) described by Dellaire and colleagues has been expanded to include a larger conserved motif apparent when additional PRP4K orthologs are taken into account (MI-2021).

The pre-mRNA processing factor 4 kinase (PRP4K) encoded by the *PRPF4B* gene, is a member of the Clk/Sty family of kinases (Corkery et al., 2015a). The kinase was first identified in a genetic screen in the fission yeast *Schizosaccharomyces pombe* and identified as Prp4 among a group of temperature sensitive mutants exhibiting splicing defects (Rosenberg, Alahari, & Käufer, 1991; Alahari et al., 1993). Alahari et al. (1993) designated this mutant Prp4 kinase allele as *prp4* ^(*ts*)^, which at the restrictive temperature (36°C) accumulated unspliced pre-mRNA and exhibited marked degradation of spliced mRNA (Alahari et al., 1993). Orthologs of PRP4K can be found across many phyla (Figure 1, and discussed below), and are characterized by a C-terminal dual-specificity kinase domain and an N-terminus containing lysine-histidine-rich (KKHK) and arginine-serine (RS)-rich protein domains (Dellaire et al., 2002); the latter domain is a common feature found among splicing proteins (Sanford et al., 2005; Shepard & Hertel, 2009; Howard & Sanford, 2015). Across these species, loss-of-function alleles of PRP4K found in plants, invertebrates (worms and fruit flies) and human cells (discussed in detail below) are typically associated with splicing defects and complete loss is lethal in several animals, indicating that PRP4K is an essential kinase in most species (Dellaire et al., 2002). Non-complementing loss-of-function alleles of *prp4* when overexpressed in fission yeast were also shown to impair mitosis (Gross et al., 1997). Later in a large scale small interfering RNA (siRNA) screen in *Drosophila melanogaster*, depletion of Prp4k was also found to induce mitotic defects (Kiger et al., 2003). Therefore, even in the earliest studies of PRP4K, it was clear that not only was this splicing kinase highly conserved in evolution, but that it was functionally pleiotropic playing both splicing-related and potentially non-splicing roles in the cell. In this review, we explore the evolutionary conservation and the diverse cellular roles of the essential splicing kinase PRP4K.

## PRP4K Domain Architecture and Evolution From Amoebae to Humans

Pre-mRNA splicing machinery is conserved across many eukaryotic lineages including metazoan animals, plants and fungi such as the baker’s yeast *S. cerevisiae* (Wahl et al., 2009). However, the relative intron density varies significantly between different eukaryote species. For example, the *S. cerevisiae* genome has genes harboring introns (primarily single introns) in ∼4% of genes, while in the human genome ∼94% of the genes have at least one intron (with an average of eight introns *per* gene) (Barbosa-Morais et al., 2006; Pan et al., 2008; Plass et al., 2008). In addition, the complexity of splicing correlates to functional diversity. As species transitioned from unicellular to multicellular life, there was the incorporation of alternative splicing in different cell types, which then promoted diverse protein expression profiles within the tissues of multicellular organisms (Busch & Hertel, 2012).

Accompanying the emergence of splicing as an important part of metazoan gene regulation was the expansion of the splicing machinery over the course of Opisthokonta evolution; the clade comprised of fungi and animals. There has been selective expansion of splicing factor families, such as the Heterogenous nuclear Ribonucleoprotein (HnRNP) proteins and SR proteins (Barbosa-Morais et al., 2006), as well as splicing kinases (Corkery et al., 2015a). Mammalian PRP4K is characterized by a C-terminal dual-specificity kinase domain and an N-terminus containing lysine-histidine-rich (KKHK) and arginine-serine (RS)-rich protein domains (Figure 1A), as well as two conserved motifs originally described as MI (DDMFA) and MII (DNWTDAEGYYRV) adjacent to the kinase domain (Figures 1B,C) (Dellaire et al., 2002). Taking into account the sequence of additional PRP4K orthologs from more species, MI can be expanded to include additional conserved sequences as DMFT(A)E(D)S.DDMFAA and is most highly conserved in vertebrates; whereas, MII is highly conserved in all PRP4K orthologs (Figure 1B). The MI and MII motifs have yet to be characterized in terms of how they contribute to PRP4K function. This is in contrast to the highly conserved kinase domain, whose function in splicing among the PRP4K orthologs has been well characterized in a variety of model organisms (Schneider et al., 2010; Eckert et al., 2016; Gao et al., 2016; Shakhmantsir et al., 2018).

Across species, the length of protein sequence encoded by orthologous PRP4K genes appears to have expanded over the course of animal evolution, and in particular the RS and KKHK domains of this kinase (Dellaire et al., 2002; Figure 1). The PRP4K kinase domain is related to the dual-specificity tyrosine DYRK family of kinases, which also includes the Yak kinases, HIPKs, and DYRKs (Aranda et al., 2011). In contrast to the RS and KKHK domains, the kinase domain of PRP4K has remained relatively similar in length and composition in metazoans (Figure 1A). Both the KKHK and RS domains of PRP4K are highly disordered regions that are thought to play roles in binding affinity to nucleic acid, splicing and protein interactions (Zhu & Krainer, 2000; Leng et al., 2007). Unlike the RS domain of PRP4K, KKHK domain has not been extensively studied. However, since RS and KKHK domains are found in splicing-associated proteins, this domain likely functions to facilitate protein interactions and with the RS domain contributes to the subnuclear localization of this kinase (Dellaire et al., 2002). The ancestral PRP4K orthologs possessing an R [S/D/E] domain follow a similar evolutionary trend for proteins containing domains that are associated with phosphorylation. To mimic the charge of constitutive phosphorylation at phosphorylation sites, molecular biologists often mutate serine residues to acidic aspartate, which was first shown by Thorsness & Koshland (1987). Intriguingly, it has been described that nature also utilizes this molecular trick in reverse, evolving serine, tyrosine, and threonine phosphorylation sites from ancestral acidic glutamate and aspartate residues (Pearlman et al., 2011). This may also be true for PRP4K, where its unikont RS domain is derived from an ancestral R [D/E/S] domain. Thus, while PRP4K RS domain sequence has diverged significantly over eukaryote evolution, the phospho-regulation of this kinase via this domain has been preserved.

## Role of PRP4K in Pre-mRNA Splicing

From the earliest experiments addressing the role of Prp4 kinase in fission yeast pre-mRNA splicing, the focus was primarily on finding genetic interactions, interacting proteins and substrates of this kinase. Early work from the Kaufer group identified non-SR splicing factor Prp1 as a substrate (Schwelnus et al., 2001) using the yeast two-hybrid assay, and using the same assay, Dellaire et al. (2002) identified pre-mRNA splicing factor 6 (PRPF6) and *Suppressor-of-white apricot* (SWAP) as interacting proteins and putative substrates in mammals. PRP4K was also shown to colocalize with the splicing factor SC35 (SRSF2) in splicing speckle domains in mammalian nuclei; a colocalization that requires the N-terminal RS-domain of PRP4K (Kojima et al., 2001; Dellaire et al., 2002). Moreover, Dellaire and others also demonstrated that PRP4K is a small nuclear ribonucleoprotein (snRNP)-associated kinase that specifically copurifies with the U5 snRNP (Dellaire et al., 2002); a finding that would later lead to the first mechanistic insights into how PRP4K regulates pre-mRNA splicing.

Pre-mRNA splicing is catalyzed by the spliceosome, a protein-RNA splicing complex that consists of five snRNPs (U1, U2, U4, U5, and U6) that are combined and remodeled in various ways to form the A, B and C spliceosomal complexes during the splicing cycle (Wahl et al., 2009). PRP4K-interacting protein PRPF6 is a stably associated component of both the U5 and tri-snRNP particles (Schaffert et al., 2004). For splicing to occur, the highly regulated assembly of RNPs occurs through protein-protein, protein-RNA, RNA-RNA interactions and protein phosphorylation events (Wahl et al., 2009). The U1 and U2 snRNPs interact with the 5′ splice site of the pre-mRNA to form the spliceosome A complex, which then forms an inactive pre-catalytic B complex through interaction with U4/U6-U5 tri-snRNP, a critical building block of the human spliceosome. Aside from the U4, U6, and U5 snRNAs, 30 distinct proteins have been identified in purified tri-snRNP complexes in mammalian cells, some of which play key roles in tri-snRNP formation and integration into the spliceosome. Building on the findings of Dellaire et al. (2002) that PRP4K interacts with PRPF6, the Luhrman group determined that PRP4K could phosphorylate the spliceosome associated proteins PRPF31 and PRPF6; two key phospho-proteins in the human spliceosomal B complex (Schneider et al., 2010). Schneider et al. (2010) further determined that phosphorylation of PRPF6 and PRPF31 by PRP4K, was a key step in tri-snRNP integration into the spliceosome and activation of the spliceosomal B complex. However, the tri-snRNPs can still dock with spliceosomal A complexes in PRP4K-depleted extracts, thus indicating that PRP4K is not required for splicing initiation and tri-snRNP docking, but rather the stable integration of the tri-snRNP during B complex formation. This role in activation of the spliceosome appears to be conserved through evolution, as *S. pombe* Prp4 kinase also interacts and phosphorylates the yeast PRPF6 ortholog, Prp1 (Schwelnus et al., 2001) and genetically interacts with the U5 snRNP proteins Prp8 and Brr2, which together promote spliceosome activation in fission yeast (Bottner et al., 2005). Given the critical role of PRP4K in activation of the spliceosomal B complex, it is perhaps unsurprising that the *PRPF4B* gene encoding PRP4K has been demonstrated to be essential in worms and in various mammalian cell lines (Dellaire et al., 2002; Hart et al., 2015).

PRP4K has also been shown to interact with and phosphorylate the SR protein SRSF1 (also referred to as SF2/ASF) (Gross et al., 1997; Kojima et al., 2001). SRSF1 phosphorylation is required to mediate 5′ splice site selection, and for regulating splice site selection during alternative splicing (Goncalves et al., 2014). The complexity and diversity of gene products encoded by the human genome arises from the fact that ∼90% of human genes having alternative splice isoforms (Pan et al., 2008; Wang et al., 2008). The most prevalent form of alternative spliced sequences are the cassette exons, which are either included or skipped during pre-mRNA splicing. Other mechanism of alternative splicing includes the use of alternative 3′ and 5′ splice sites or intron retention (reviewed in El Marabti & Younis, 2018). Most often, these events are regulated by SR proteins through RNA recognition motif-mediated binding to exonic splicing enhancers to mediate splice site selection (Lin & Fu, 2007). Phosphorylation of SR proteins can promote both their nucleo-cytoplasmic shuttling via the specialized transportin-SR (also known as TNPO3) and their nuclear localization to and from splicing speckles, thereby regulating the rate in which SR proteins bind target transcripts (Kataoka, Bachorik, & Dreyfuss, 1999; Lin & Fu, 2007). Since PRP4K phosphorylates SRSF1 and likely other SR proteins, it is possible that PRP4K may also affect pre-mRNA splicing by modulating SR-protein shuttling and/or protein-RNA interactions to affect splice site selection. Indeed, the Kaufer group has demonstrated that chemical inhibition of Prp4 kinase activity results in selective misplicing of weak exon 1/5’ splicing events in fission yeast (Eckert et al., 2016).

## PRP4K as a Regulator of Transcription and Cell Signaling

In addition to regulating pre-mRNA splicing, the N-terminus of PRP4K has been shown to interact with proteins involved in chromatin remodeling and regulation of gene transcription (Dellaire et al., 2002). For example, this N-terminal region of PRP4K interacts with BRG1 (Dellaire et al., 2002), a mammalian homologue of the *Drosophila* protein Brahma and the catalytic component of a mammalian Swi/Snf complex involved in the maintenance of homeotic gene regulation (Dingwall et al., 1995). Specifically, Dellaire et al. (2002) found that PRP4K and BRG1 interacted in a transcription-dependent manner, supporting a potential role for PRP4K in chromatin remodeling events during co-transcriptional splicing. Another chromatin-associated factor identified as a PRP4K-interacting protein is the nuclear receptor corepressor 1 (NCOR1), a protein that represses basal transcription through the direct or indirect recruitment of histone deacetylases (HDACs; Underhill et al., 2000). NCOR1 is found in two biochemically distinct complexes, N-CoR-1 and N-CoR-2. The N-CoR-2 complex was revealed to contain PRP4K along with deacetylase components, and purification of a megadalton PRP4K/N-CoR complex from mammalian cells containing BRG1 also exhibited strong deacetylase activity (Dellaire et al., 2002). Therefore, it is possible that the PRP4K/N-CoR-2 and BRG1 containing complex may coordinate both gene repression as well as co-transcriptional pre-mRNA splicing.

PRP4K is also likely a mediator of the crosstalk between transcription and splicing by phosphorylating both SR proteins and transcription factors. For example, PRP4K can phosphorylate and regulate both the T-cell transcription factor Krüppel-like factor 13 (KLF13) and the ETS transcription factor 1 (ELK1) (Huang et al., 2000; Huang et al., 2007). In the case of the latter, stimuli such as the epidermal growth factor (EGF) or forskolin, activate PRP4K to then phosphorylate ELK1 on Thr-417 (Huang et al., 2000). While ELK1 is the target of MAPK phosphorylation at Ser-383 in response to EGF stimulation, the phosphorylation of ELK1 at Thr-417 by PRP4K appears to be unique to this kinase.

The Thr-417 phosphorylation event has consequences for both neurodegeneration and cancer. For example, ELK1 Thr-417 phosphorylation triggers apoptosis in neurons and in neurodegenerative disease such as Lewy body disease, Alzheimer’s disease and Huntington’s disease, and phosphorylated ELK1 (positive for Thr-417) is found in the different protein inclusions/aggregates associated with these disease (Sharma et al., 2010). In regard to cancer, the phosphorylation of this threonine residue in ELK1 is associated with a number of malignancies, including colonic adenocarcinomas where Thr-417 phosphorylation is significantly elevated in this carcinoma compared to normal colonic epithelium (Morris et al., 2013). Therefore, while PRP4K regulation of ELK1 demonstrates its role in connecting splicing and transcriptional machinery, there are potential unexplored clinical ramifications of the phosphorylation of ELK1 by PRP4K for both neurodegeneration and cancer. We will explore the links between PRP4K and cancer in the next sections, beginning with the role of PRP4K in regulating cell division and the cellular response to taxane-based chemotherapy.

## PRP4K Regulates Cell Division via the Spindle Assembly Checkpoint

**FIGURE 2:**
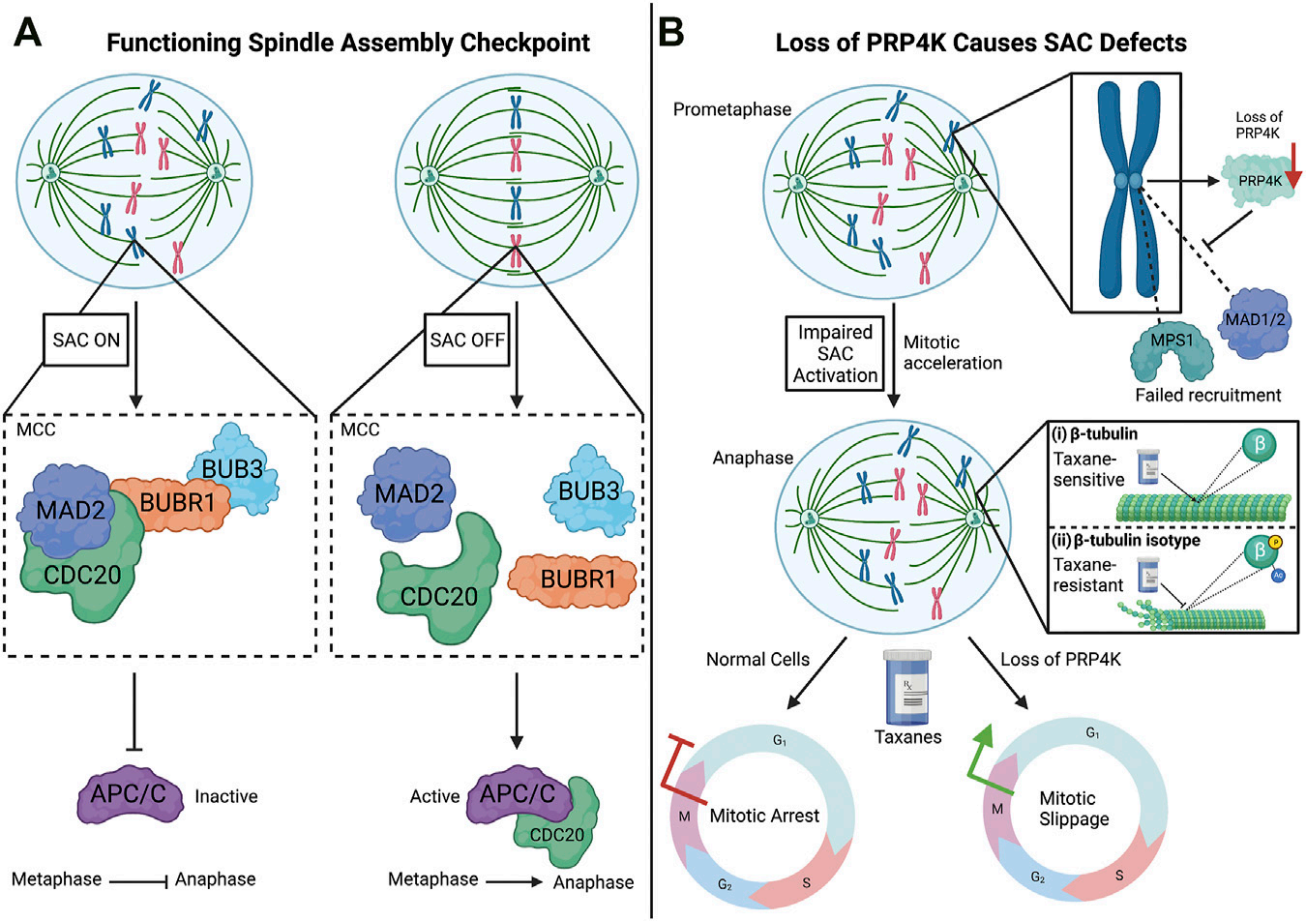
The spindle assembly checkpoint (SAC) and impact of PRP4K loss on the SAC. **(A)** SAC is activated when chromosomes are not properly attached to the spindle microtubules through their kinetochores. This activation is dependent on the recruitment of the SAC proteins, MAD2, CDC20, BUBR1, and BUB3, which form the mitotic checkpoint complex (MCC) at kinetochores. The binding of SAC target CDC20 to the MCC prevents its association with APC/C (anaphase-promoting complex/cyclosome), which promotes the transition from metaphase to anaphase. When chromosomes are properly aligned, the assembly of the MCC is inhibited turning off the SAC, which in turn triggers APC/C to associate with CDC20 and promote the onset of anaphase. **(B)** Loss of PRP4K impairs SAC activation by anti-mitotic agents such as the taxanes. Normal cells treated with taxanes (anti-mitotic agent) results in mitotic arrest during the mitotic (M) phase, but cells with low levels of PRP4K fail to activate the SAC and undergo mitotic slippage into G1 without chromosome segregation. Mechanistically, loss of PRP4K contributes to taxane resistance by impairing drug-induced recruitment of SAC proteins MPS1 and MAD1/2 to kinetochores to trigger the SAC. In normal cells, taxanes bind the β-tubulin subunit of microtubules and induce dynamic stability, leading to cell death (i). However, both direct post-translational modification (e.g., phosphorylation or acetylation) of β-tubulin subunits or changes in tubulin isoforms expression can alter taxane binding and contribute to taxane resistance by preventing microtubule stabilization by these drugs (ii). Elements of this figure were created with BioRender.com.

To prevent chromosome instability (CIN) during mitosis and meiosis, the spindle assembly checkpoint (SAC) acts to delay the final stages of cell division until chromosomes are attached to microtubule spindles and aligned at the spindle equator (reviewed in Lara-Gonzalez et al., 2012) (Figure 2A). More specifically in mitosis, anaphase is delayed by the SAC until chromosomes are properly aligned and the SAC is inactivated, and this regulatory mechanism is essential to avoid chromosome mis-segregation and aneuploidy, which has been implicated in tumorigenesis (Chi et al., 2009; Li et al., 2009). However, for proper spindle function, tension must be maintained between the spindle poles and the chromosomes by continuous polymerization at the kinetochore end of the microtubule attached to each chromosome and depolymerization at the spindle poles (Wittmann et al., 2001). Consequently, microtubule poisons that either destabilize microtubules such as vincristine or aberrantly stabilize microtubule such as the taxane paclitaxel are used to treat cancer by triggering the SAC, which in turn triggers mitotic catastrophe and cell death in rapidly dividing cancer cells (Mukhtar et al., 2014; Yasuhira et al., 2016).

SAC activation is dependent upon the hierarchical recruitment of regulatory proteins to kinetochores during early stages of mitosis (Figure 2A). In the early 1990s, several of these proteins were identified that contribute to the maintenance and surveillance of chromosome segregation in yeast, including Mad1, Mad2, Mad3 (mitotic-arrest deficient), Bub1, Bub3 (budding uninhibited by benzimidazole), and Mps1 (multipolar spindle-1) (Hoyt et al., 1991; Li & Murray, 1991; Weiss & Winey, 1996). Liu et al. (2006) would then go on to show the hierarchical recruitment and localization of these SAC proteins to kinetochores. For example, the localization of MPS1 (also known as TTK) at kinetochores is dependent upon the centromere protein (CENP)-I, which in turn recruits MAD1 (MAD1L1) and MAD2 (MAD2L1) (Liu et al., 2006). The signaling activity of the SAC persists until the mitotic checkpoint complex (MCC), which contains MAD2, BUBR1, and BUB3, becomes bound to the SAC target CDC20. CDC20 is an activator of the multisubunit E2 ubiquitin ligase anaphase-promoting complex/cyclosome (APC/C), which promotes the transition from metaphase to anaphase. In the event where kinetochores are unattached, the SAC is activated, which in turn inhibits CDC20 and prevents transition from metaphase to anaphase (Kramer et al., 1998). Conversely, the attachment of kinetochores to spindle microtubules triggers APC/C activation to ubiquitinate mitotic proteins to target their degradation via the proteasome, thus allowing mitotic progression (Alexandru et al., 1999).

A putative role for PRP4K in cell division was first suggested by mitotic defects seen in *S. pombe* expressing a dominant truncated Prp4 mutant (Gross et al., 1997). Later in the fly *D. melanogaster* depletion of the ortholog of PRP4K (Dmel/Prp4k/CG7028) was found to impair mitosis (Kiger et al., 2003). In addition, Dellaire and others demonstrated that on isolated mitotic chromosomes, PRP4K could be detected along chromosome arms including at the kinetochores (Dellaire et al., 2002). Taking these findings into account, Montembault et al. (2007) investigated PRP4K in the context of mitosis. The authors reported that PRP4K associates with kinetochores during mitosis, and that the depletion of the protein promoted mitotic acceleration, a similar phenotype shown in the absence of Mad2 or BubR1 (Meraldi et al., 2004). In addition, PRP4K-depleted cells in anaphase and telophase had lagging chromatids/chromosomes, failed to arrest in mitosis when treated with the microtubule-targeting drug noncodazole, and re-entered interphase without chromosome segregation in drug-treated cells (Montembault et al., 2007). Since noncodazole triggers the SAC in normal HeLa cells through microtubule depolymerization, components of the SAC were analyzed in control and PRP4K-depleted cells to determine the mechanism by which PRP4K loss triggered failure of the SAC. This analysis revealed that MPS1, MAD1, and MAD2, normally recruited to kinetochores to trigger the SAC in response to noncodazole, did not localize to the kinetochores when PRP4K was depleted. Together these data indicate that PRP4K is a key regulator of the SAC and its depletion likely contributes to CIN and aneuploidy (summarized in Figure 2), major drivers of cancer development and evolution (Sansregret & Swanton, 2017). However, since these studies did not examine a kinase dead form of PRP4K, it remains unknown whether its ability to regulate SAC is kinase-dependent. Furthermore, although assumed that localization of PRP4K to kinetochores during mitosis is dependent on components of the SAC, such as MAD1/MAD2/BUBR1, to date no specific protein-protein interaction has been identified that mediates this localization. Such a CIN phenotype could also lead to impaired tumour growth, at least initially. For example, trifluridine-induced CIN was shown to impair triple negative breast cancer (TNBC) tumour growth (Li et al., 2020), and at least one study has linked depletion of PRP4K in the MDA-MB-231 xenograft model of TNBC to reduced metastasis in mice (Koedoot et al., 2019). In the next section, we explore more deeply the experimental evidence linking PRP4K regulation to cancer development and therapy responses.

## PRP4K as a Cancer Biomarker and Haploinsufficient Tumour Suppressor

### PRP4K is a Biomarker for Taxane Sensitivity in Breast and Ovarian Cancer

PRP4K protein expression is highly variable in tumour cells from various cancers, and reduced PRP4K expression in breast and ovarian cancer correlates with worse overall survival in several studies (Corkery et al., 2015b; Cho et al., 2018; Corkery et al., 2018). Taxanes such as paclitaxel and docetaxel are used to treat many different cancers including breast and ovarian cancer, and kill cancer cells by disrupting microtubule dynamics, triggering the SAC followed by mitotic arrest and cell death (Yasuhira et al., 2016) (Figure 2B).

Common mechanisms of taxane resistance encountered *in vitro* are either upregulation of the major efflux pump for taxanes, the multi-drug resistance 1 (MDR1) gene (also known as p-glycoprotein), and/or direct modification of microtubules themselves, triggered by tubulin isotype selection and/or post-translational modifications of tubulin subunits that alter regulatory protein and drug binding (Orr, Verdier-Pinard, McDaid, & Horwitz, 2003) (Figure 2B). Clinically, the expression of the human epidermal growth factor receptor 2 (HER2, also known as the erb-b2 receptor tyrosine kinase ERBB2) has been linked to taxane therapy responses in breast cancer patients (Perez et al., 2005). HER2/ERBB2 is overexpressed in 20–30% of breast cancer cases (Wahler & Suh, 2015). Its prevalence in breast cancer has made it a widely studied biomarker for patient taxane response, such that its amplification *in vitro* was shown in one study to promote increased resistance to taxanes (Yu et al., 1996). However, use of HER2/ERBB2 as a biomarker for taxane response remains controversial, as other studies have shown that its amplification correlates with better clinical response to taxanes (Di Leo et al., 2004). This apparent contradiction in the literature may arise due additional heterogeneity of HER2 positive breast cancer, and therefore sensitivity of a given patient’s tumour to taxane may rely on additional genetic modifiers of treatment response. This prompted Corkery and others to examine more deeply the relationship between PRP4K gene regulation and taxane resistance in breast and ovarian cancer (Corkery et al., 2015b).

Corkery et al. (2015a) identified PRP4K as a novel HER2-regulated protein in breast and ovarian cancer that when depleted could reduce the sensitivity of breast and ovarian cancer cells to the taxane paclitaxel. Moreover, they found that among a taxane-treated cohort of ovarian cancer patients, the patients harboring tumours with high PRP4K protein levels, and low levels of HER2 expression, exhibited better overall survival (Corkery et al., 2015b); evidence supporting a potential role for PRP4K as a predictive biomarker for taxane response in ovarian cancer. Furthermore, ovarian carcinoma cells isolated from the ascites of a relapsed ovarian cancer patient initially responsive to taxanes, exhibited markedly reduced PRP4K expression compared to the primary tumour and correlated with taxane resistant disease (Corkery et al., 2015b). In a separate study, PRP4K was also identified as an estrogen-regulated gene, and that treatment of cells with 4-hydroxy-tamoxifen (4-OHT), the active metabolite of the anti-estrogen drug tamoxifen, resulted in both reduced PRP4K expression and an increase in taxane resistance in MCF7 breast cancer cells expressing the estrogen receptor 1 (ESR1) (Lahsaee et al., 2016). As discussed previously, PRP4K expression is required for effective regulation of the SAC, and its loss prevents mitotic arrest after the use of anti-mitotic agents (Montembault et al., 2007). Consistent with these findings, Corkery and others found that depletion of PRP4K in multiple breast and ovarian cancer cell lines resulted in decreased sensitivity to paclitaxel, and whereas control cells arrested in metaphase and underwent apoptosis, PRP4K-depleted cells underwent “mitotic slippage” entering interphase without cell division (Corkery et al., 2015b).

### PRP4K Contributes to Tumour Suppression by Regulating Anoikis

**FIGURE 3:**
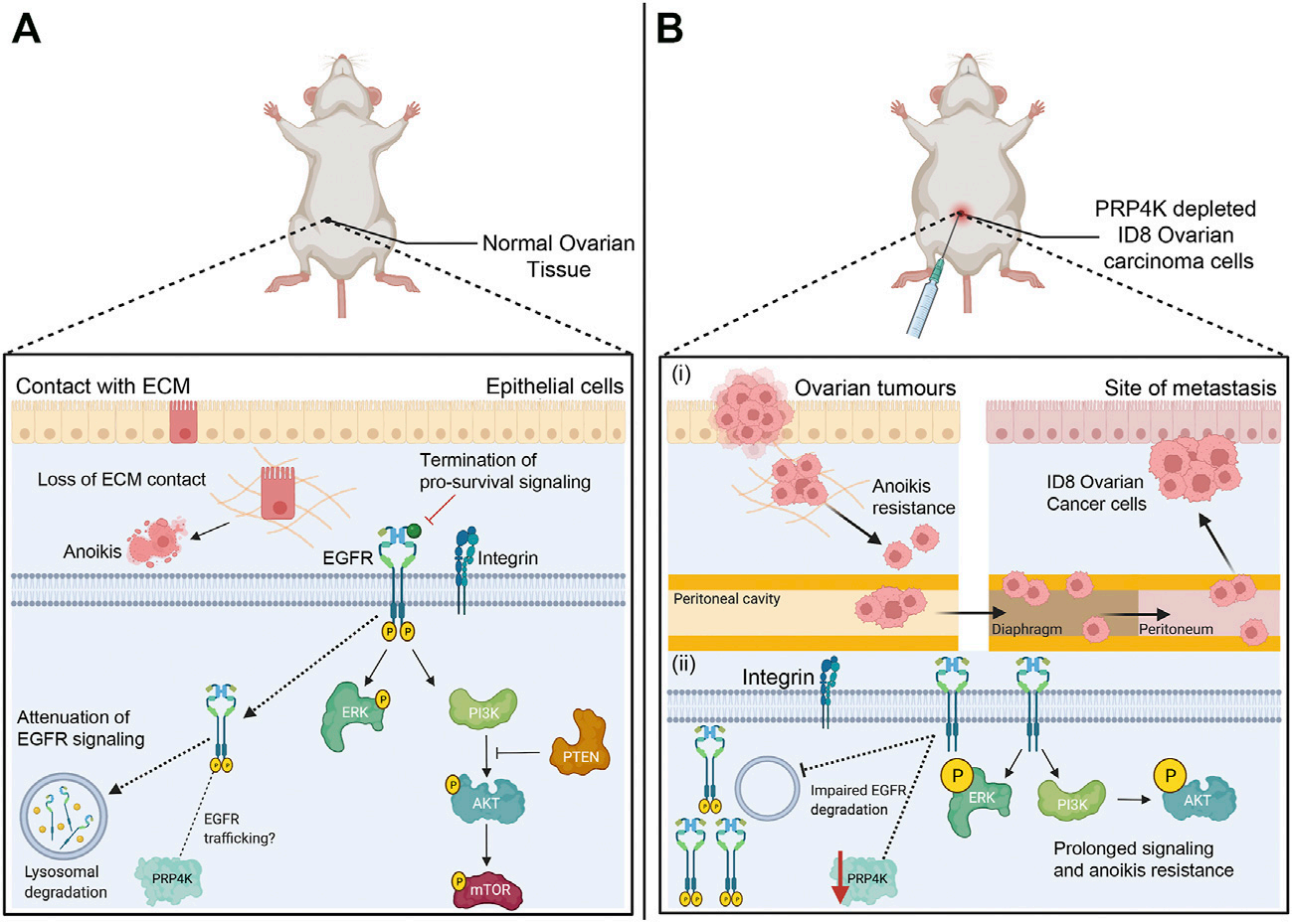
Anoikis pathway is a barrier against metastasis and loss of PRP4K triggers anoikis resistance by promoting pro-survival signaling. **(A)** Anoikis pathway induces cell death (apoptosis) after cell detachment from the ECM (extracellular matrix) to prevent adherent-independent cell growth and attachment. Pro-survival pathways that regulate cell survival and proliferation are terminated once integrins disengage from the ECM. Epithelial growth factor receptor (EGFR) signaling is one such pathway, and detachment triggers EGFR trafficking to the lysosome and its degradation to attenuate of EGFR signaling; a feedback loop that is dependent upon the presence of PRP4K. EGFR degradation consequently results in decreased ERK and PI3K/AKT/mTOR kinase activation to further suppress pro-survival signaling. In addition, PTEN (phosphatase-tensin) acts as a tumour suppressor to inhibit growth by negatively regulating the PI3K/AKT/mTOR pathway. **(B)** The depletion of PRP4K leads to anoikis resistance and cell survival in xenotransplanted mouse ID8 ovarian carcinoma cells under detached growth conditions. PRP4K depleted ID8 cells disseminate within the peritoneal cavity which then promote anchorage-independent growth and metastasis in the diaphragm and peritoneum. Loss of PRP4K results in impaired degradation of EGFR, which in turn results in sustained ERK and PI3K/AKT kinase activation to promote cell survival and anoikis resistance in PRP4K-depleted cells. Elements of this figure were created with BioRender.com.

Despite being a kinase that first emerged in unicellular eukaryotes, there are uniquely multicellular functions for PRP4K. One of which involves its regulation of the anoikis pathway. Anoikis is a signaling pathway that triggers cell death when cells detach from the extracellular matrix (ECM) within a tissue (Buchheit et al., 2014; Elmore, 2007; Paoli et al., 2013) (Figure 3A). Thus, this form of programmed cell death acts as a fail-safe to maintain normal tissue homeostasis following tissue damage by limiting inappropriate growth and migration of detached cells. Importantly, anoikis acts as a barrier to metastasis, as cancer cells must first survive this form of cell death during detachment in order metastasize to distant sites and tissues (Mehlen & Puisieux, 2006; Kim et al., 2012).

Corkery et al. (2018) first described the capacity for PRP4K to overcome anoikis using a novel xenotransplantation assay in zebrafish, and the ID8 mouse model of ovarian carcinoma (Corkery et al., 2018) (Figure 3B). In the zebrafish xenotransplantation assay, cells are injected into the yolk sac of fish embryos (Corkery et al., 2011), which is an environment that is acellular, nutrient rich and lacks an ECM that would otherwise prevent anoikis following transplantation of carcinoma cells. Corkery and others discovered that depletion of PRP4K allowed xenotransplanted mouse ID8 cells to survive and grow within the fish almost twice as well as control cells with normal levels of PRP4K (Corkery et al., 2018). PRP4K depleted ID8 cells and human MCF7 breast cancer cells also exhibited greater anchorage-independent growth and reduced apoptosis when grown in suspension, consistent with reduced sensitivity to anoikis. When ID8 ovarian cancer cells were transplanted into mice and the surviving fraction of cells was assessed 28 days later, PRP4K expression was greatly reduced, suggesting a survival advantage with PRP4K loss. Finally, depletion of PRP4K in ID8 cells prior to their transplantation resulted in a 2-fold increase in metastasis within the peritoneal cavity of the mice compared to controls (Corkery et al., 2018). Thus, reduced PRP4K expression promotes anchorage-independent growth and metastasis in ovarian cancer.

To understand how PRP4K loss contributes to increased anoikis resistance, we first have to understand the inter-relationship between pathways involved in cell adhesion and growth factor signaling. Cell surface integrins bind to the ECM and form focal adhesions, which are large multi-protein complexes that act as the link between sensing mechanical changes and intracellular signaling that promotes cell survival, migration, and proliferation (Buchheit et al., 2014; Elmore, 2007; Paoli et al., 2013). Receptor tyrosine kinases, including EGFR, are major contributors to focal adhesion signaling (Javadi et al., 2020). Once integrin disengagement occurs, EGFR is trafficked to the lysosome for degradation, and this terminates pro-survival signaling that would otherwise promote cell survival under detached growth conditions (Grassian et al., 2011; Reginato et al., 2003). Within this important growth factor feedback loop linked to anoikis, PRP4K is a required component, and depletion of PRP4K leads to impaired degradation of EGFR and sustained EGFR signaling (Corkery et al., 2018) (Figure 3B). Once initiated, prolonged EGFR signaling resulted in sustained ERK and AKT kinase activation in PRP4K depleted cells that promoted cell survival and resistance to anoikis. These data are reminiscent of the effects of PTEN depletion, which activates ERK and AKT signaling to promote anoikis resistance (Vitolo et al., 2009). PTEN is a haploinsufficient tumour suppressor that inhibits growth signaling by dephosphorylating phosphatidylinositol 3,4,5-trisphosphate (PIP3), the product phosphatidylinositol 3-kinase (PI3K) that in turn activates AKT (Georgescu, 2010). Like PRP4K, reduced PTEN gene expression is sufficient to promote increased AKT signaling and consequently tumour development (Alimonti et al., 2010). Therefore, in the context of epithelial cancers, PRP4K behaves as a haploinsufficient tumour suppressor whose depletion enhances EGFR signaling and anoikis resistance.

### PRP4K Regulates Yap Signaling and is Negatively Regulated During EMT

**FIGURE 4:**
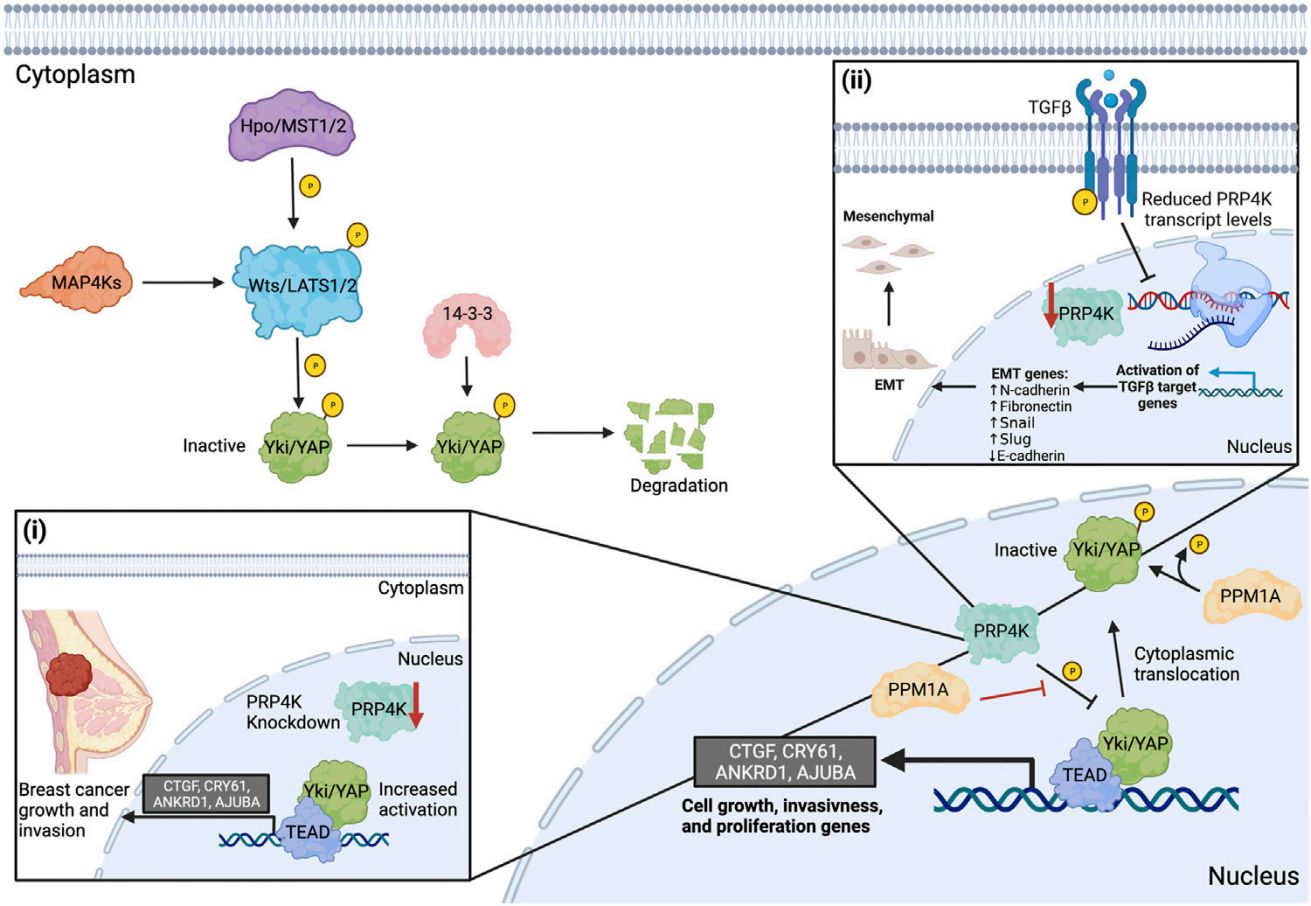
PRP4K is a negative regulator of Hippo-Yap signaling and EMT reduces PRP4K expression in a feed-forward loop to promote aggressive tumour growth and invasiveness. The upstream kinase Hpo/MST1/2 (Ste20 family kinases) phosphorylates and activates Wts/LATS1/2 (Nuclear Dbf-2-related kinase family) downstream. MAP4K family members act in parallel with Hpo/MST1/2 to regulate Wts/LATS1/2. Wts/Lats1/2 phosphorylates Yki/YAP to inactivate it and recruitments 14-3-3 to promote its cytoplasmic retention and degradation. In its dephosphorylated state (active), YAP translocates to the nucleus and interacts with TEAD family transcription factors (TEAD1-4) to activate target genes (CTGF, CRY61, ANKRD1, AJUBA) that regulate tumour cell growth, invasiveness, and migration. In the nucleus, PRP4K negatively regulates Yki/YAP signaling. PRP4K directly inhibits the binding of Yki/YAP to TEAD, preventing the expression of target genes. To prevent the phosphorylation of YAP, PPM1A suppresses PRP4K phosphorylation or dephosphorylates Yki/YAP in the nucleus. Loss of PRP4K prevents the phosphorylation and nuclear exit of Yki/YAP, leading to increased activation of target genes that promote breast cancer cell growth and invasion (i). Induction of epithelial-to-mesenchymal transition (EMT), for example in response to TGFβ, results in both increased expression of genes that promote cancer growth and metastasis and reduced PRP4K expression, the latter forming a feed-forward loop to further promote aggressive cancer growth and metastasis by activating Yap signaling (ii). Elements of this figure were created with BioRender.com.

During development, organs eventually reach a final size and growth is restricted. The regulated interplay between cell proliferation and cell death determines organ size. Organism-intrinsic pathways exist to limit organ growth even in the abundance of nutrients and growth hormones. One of the main regulatory pathways controlling tissue growth is the Hippo-Yes-associate protein (Yap) signaling pathway (Yu et al., 2015) (Figure 4). Owing to its role in maintaining tissue homeostasis, this pathway is often dysregulated in numerous malignancies (Calses et al., 2019; Yu et al., 2015; Zanconato et al., 2016). The core regulatory machinery of Yap signaling consists of two kinase families that are highly conserved from *Drosophila* to vertebrates (Snigdha et al., 2019). These kinases include the upstream Hpo/MST1/2 (Ste20 family kinases) and the downstream kinases they phosphorylate and activate, Wts/LATS1/2 (Nuclear Dbf-2-related kinase family), which in turn phosphorylate Yki/YAP to inhibit Yap signaling (Yu et al., 2015) (Figure 4). The phosphorylation of YAP leads to it being excluded from the nucleus, in part due to 14-3-3 recruitment, which sequesters phosphorylated YAP in the cytoplasm where it is degraded by the proteasome (Oh & Irvine, 2008; Pan, 2010; Ren et al., 2010). In the absence of a phosphorylation event, YAP translocates to the nucleus and interacts with TEAD family transcription factors (TEAD1-4) to promote the expression of target genes that regulate cell growth, proliferation, and survival (Holden & Cunningham, 2018).

A connection between YAP and PRP4K was recently uncovered by Cho et al. (2018) in *Drosophila melanogaster,* where a genetic screen uncovered the fly ortholog of PRP4K (Dmel/Prp4k) as a new regulator of Hippo-Yap signaling. Cho and others found that knockdown of Prp4k in a fly mutant overexpressing the Yorkie (Yki, the fly ortholog of YAP) exaggerated the overgrowth phenotype observed in mutant eyes. Conversely, overexpression of Prp4k in the Yki mutant fly suppressed the overgrowth phenotype, indicating that Prp4k negatively regulates Yap signaling. The regulation of Yap signaling is dependent on the kinase activity of Prp4k, since the overexpression of a kinase dead form of Prp4k failed to suppress the overgrowth phenotype (Cho et al., 2018). It was then determined that Prp4k phosphorylates Yki at Ser111 and Ser250 in the nucleus. These data indicate that in addition to cytoplasmic 14-3-3, other molecular players exist to promote YAP translocation out of the nucleus. Since Lats1/2 shares a Yki phosphorylation site with Prp4k at Ser250, it is also likely these two kinases work together to promote cytoplasmic Yki localization and inactivation (Figure 4).

Several studies have now demonstrated that PRP4K regulation of YAP is a conserved process between flies and mammals (Cho et al., 2018; Clarke et al., 2021; Zhou et al., 2021). For example, Clarke et al. (2021) showed that when EMT was induced by media containing transforming growth factor beta (TGFβ) or as a result of EIF3E loss in the non-transformed mammary cell line MCF10A, PRP4K expression was reduced (Figure 4), and this correlated with increased YAP nuclear translocation and target gene expression in EIF3E-depleted MCF10A cells. Intriguingly, activation of Yap signaling seemed to occur as a result of PRP4K loss, since the addback of PRP4K in EIF3E-depleted cells led to increased YAP shuttling to the cytoplasm and reduced YAP target gene expression. Thus, PRP4K expression status is likely a modifier of YAP signaling that could represent a “feed-forward” loop to further promote EMT in malignant cells. In light of these findings, we are lead to pose another important question: If PRP4K acts as the nuclear kinase promoting YAP shuttling to the cytoplasm to “turn-off” the nuclear activities of YAP, are there phosphatases that prevent the phosphorylation of YAP within the nucleus?

One possible candidate phosphatase affecting YAP phosphorylation in the nucleus is PPM1A (Zhou et al., 2021). Zhou et al. (2021) showed through immunoprecipitation experiments that PPM1A directly dephosphorylated YAP and that this occurred within the nuclei of regenerating mammalian intestinal and liver cells. Furthermore, YAP activity could be inhibited when PRP4K was transiently overexpressed in human HEK293 cells alone, but co-expression with exogenous PPM1A counteracted the inhibition of YAP by PRP4K. Thus, in mammals, while MST1 and LATS1/2 regulate YAP in the cytoplasm, the PRP4K-PPM1A axis regulates YAP phosphorylation in the nucleus. As such, this may represent an important physiological regulatory mechanism controlling Yap signaling during liver and intestinal regeneration (Zhou et al., 2021). However, in malignant cells, aberrant Yap signaling is associated with increased tumour cell growth, migration, and invasion (Cho et al., 2018; Clarke et al., 2021). Cho et al. (2018) found that PRP4K knockdown promotes breast cancer cell growth and invasion, in part facilitated by the increased activation of YAP target genes (e.g. CTGF, CRY61, ANKRD1 and AJUBA) (Figure 4). Similarly, Clarke et al. (2021) found that PRP4K knockdown promotes transformed breast cancer cell migration and invasion, yet appeared to inhibit 2D cell migration in non-transformed MCF10A cells. Thus, transformation state of the cell may play an important role in physiological outcome of PRP4K depletion in regard to enhanced cell migration and invasion. Nonetheless, together these data support a role for PRP4K as a key negative regulator of Yap both during normal physiological processes such as organ regeneration, as well as during malignant transformation.

## Concluding Remarks

**FIGURE 5:**
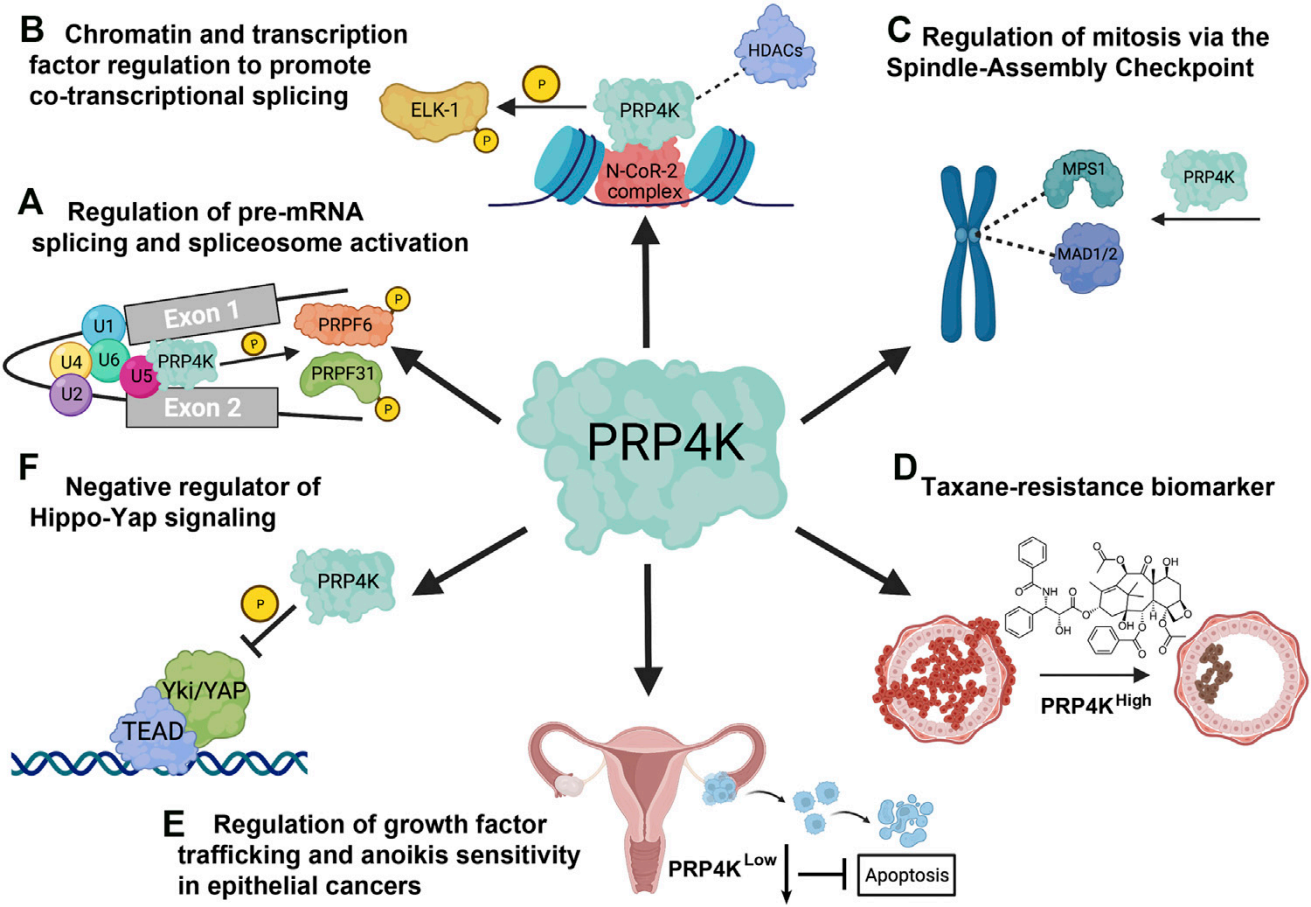
Overview of the cellular functions of PRP4K. **(A)** PRP4K was originally identified for its role in pre-mRNA splicing and is a component of the U5 snRNP that phosphorylates spliceosome associated proteins PRPF31 and PRPF6, a key step in tri-snRNP integration and spliceosomal B complex activation. **(B)** PRP4K is implicated in gene regulation both through association with chromatin remodeling proteins such as the N-CoR-2 (nuclear receptor corepressor) complex containing BRG1 and histone deacetylases (HDACs), and transcription factors such as ELK-1 that are substrates of PRP4K. ELK-1 phosphorylation by PRP4K may play a role in development of certain cancers, including colorectal cancer. **(C)** PRP4K regulates the spindle assembly checkpoint (SAC) by recruiting proteins MPS1 and MAD1/2 to kinetochores for effective mitotic progression. Consequently, the SAC function is impaired in PRP4K-depleted cells, resulting in chromosome mis-segregation and aneuploidy. **(D)** PRP4K is a biomarker for taxane resistance as breast and ovarian cancers with reduced PRP4K expression are more resistant to taxanes; a phenotype that is likely a result of impaired SAC activation and failed mitotic arrest in drug-treated cells. **(E)** PRP4K regulates anoikis sensitivity by contributing to the attenuation of growth factor signaling when cells detach from the extracellular matrix (ECM). Consequently, when PRP4K expression is low it impairs trafficking and degradation of growth factor receptors such as EGFR, which in turn promotes pro-survival ERK/AKT signaling and anoikis resistance. **(F)** PRP4K negatively regulates YAP signaling by promoting its translocation from the nucleus to the cytoplasm, thereby preventing the expression of target genes that regulate cell growth and proliferation. The loss of PRP4K causes aberrant YAP signaling which is associated with uncontrolled cell proliferation, and increased cell migration in malignant cells. Elements of this figure were created with BioRender.com.

In the past decade, there have been several studies supporting the notion that the cellular role of PRP4K goes beyond pre-mRNA splicing, and that it has diverse regulatory functions in tumour suppression and chemotherapeutic responses (summarized in Figure 5). A few key studies have bridged the gap between PRP4K and its role in cancer by uncovering regulatory functions in cell division, Yap signaling and the cellular response to taxane-based chemotherapy. PRP4K acts as a key regulator of the SAC during mitosis, and thus its loss alters therapy responses to microtubule targeting chemotherapy and although not experimentally validated, likely also drives CIN during cancer development. Since PRP4K protein expression is variable in breast and ovarian cancer, PRP4K may represent a useful predictive biomarker for taxane response, particularly following relapse in ovarian cancer patients treated with taxanes (Corkery et al., 2018). Importantly, depletion rather than complete loss of PRP4K expression promotes not only resistance to taxanes but also more aggressive cancer phenotypes including anoikis resistance, increased cell migration and aberrant growth factor signaling. Together these data indicate that PRP4K is a haploinsufficient tumour suppressor in epithelial cancers. Despite the growing literature supporting the tumour suppressor role(s) of PRP4K, there are still many mechanistic details missing regarding how PRP4K expression, kinase activity and subcellular localization are regulated, and how low PRP4K expression contributes to cancer development. In particular, to what extent does altered pre-mRNA splicing in PRP4K low tumours contribute to their treatment responses and progression relative to altered phosphorylation of key substrates of this kinase? For example, PRP4K negatively regulates Hippo-Yap signaling through YAP phosphorylation, providing one mechanism by which reduced kinase expression could contribute to increased migration and cancer cell invasion through dysregulation of Yap-target gene expression. In addition, recent work linking induction of EMT and negative regulation of PRP4K expression provides the first evidence of dynamic regulation of PRP4K gene expression and protein translation during cancer development. Therefore, continued research on mechanisms regulating PRP4K expression, substrate specificity and kinase activity, will provide key insights into the cellular roles and tumour suppressor activities of this multi-functional kinase.
